# The Prevalence of Sacroiliitis and Spondyloarthritis in Patients with Sarcoidosis

**DOI:** 10.1155/2014/289454

**Published:** 2014-05-12

**Authors:** Senol Kobak, Fidan Sever, Ozlem Ince, Mehmet Orman

**Affiliations:** ^1^Department of Rheumatology, Faculty of Medicine, Sifa University, Bornova, 35100 Izmir, Turkey; ^2^Department of Chest Diseases, Faculty of Medicine, Sifa University, Bornova, 35100 Izmir, Turkey; ^3^Department of Radiology, Faculty of Medicine, Sifa University, Bornova, 35100 Izmir, Turkey; ^4^Department of Statistic, Faculty of Medicine, Ege University, Turkey

## Abstract

*Introduction.* Sarcoidosis is a chronic granulomatous disease, which can involve different organs and systems. Coexistence of sarcoidosis and spondyloarthritis has been reported in numerous case reports. *Purpose.* To determine the prevalence of sacroiliitis and spondyloarthritis in patients previously diagnosed with sarcoidosis and to investigate any possible relation with clinical findings. *Materials and Methods.* Forty-two patients with sarcoidosis were enrolled in the study. Any signs and symptoms in regard to spondyloarthritis (i.e., existence of inflammatory back pain, gluteal pain, uveitis, enthesitis, dactylitis, inflammatory bowel disease, and psoriasis) were questioned in detail and biochemical tests were evaluated. Sacroiliac joint imaging and lateral heel imaging were performed in all patients. *Results.* Sacroiliitis was found in 6 of the 42 (14.3%) sarcoidosis patients and all of these patients were female. Common features of the disease in these six patients were inflammatory back pain as the major clinical complaint, stage 2 sacroiliitis as revealed by radiological staging, and the negativity of HLA B-27 test. These six patients with sacroiliitis were diagnosed with spondyloarthritis according to the criteria of ASAS and of ESSG. *Conclusion.* We found spondyloarthritis in patients with sarcoidosis at a higher percentage rate than in the general population (1–1.9%). Controlled trials involving large series of patients are required for the confirmation of the data.

## 1. Introduction

Sarcoidosis is a systemic disease characterized by the involvement of multiple tissues and organs with a noncalcified granuloma reaction, which is not yet well understood [1]. Although the exact pathogenesis of sarcoidosis is not known, it is currently accepted that, in genetically susceptible individuals, it is caused through alteration of the cellular immune response after exposure to an environmental, occupational, or infectious agent [2]. Accumulations of Th1 and macrophages with increased production of proinflammatory cytokines induce the inflammatory cascade and consecutive impairment in tissue permeability; increase in cellular influx and local cellular proliferation cause the formation of granulomas [3]. The crucial pathological finding of sarcoidosis is noncalcified epitheloid cellular granulomas [4]. Sarcoidosis is a chronic granulomatous disease that may present with various clinical findings. It may mimic a number of primary rheumatic diseases and/or accompany them [5]. The disease most frequently presents with bilateral hilar lymphadenopathy and infiltrations in the lungs and skin, as well as with eye lesions. Locomotor involvement is also determined in 15–25% of the patients [6]. Two major joint involvement patterns are defined, acute and chronic forms. Acute disease is the most common form and the patient presents with arthralgia and signs of arthritis and/or periarthritis as the first sign of sarcoidosis. Chronic sarcoid arthritis usually coexists with pulmonary parenchymal disease or other organ involvement and is rare [7]. Spondyloarthropathies are a group of chronic inflammatory diseases primarily characterized by the involvement of axial and peripheral joints [8]. Common characteristics of these diseases are inflammatory back pain, enthesitis, uveitis, psoriasis, inflammatory bowel disease, and RF negativity. Involvement of the sacroiliac joint is one of the major findings. Coexistence of sarcoidosis and sacroiliitis has been published in numerous case reports [9]; however the prevalence of sacroiliitis and spondyloarthritis has not been reported in sarcoidosis. Evidence is collected for the association of sarcoid-like granulomatous disease developing after the initiation of anti-TNF-**α** therapy, with disease reversal after discontinuation [10]. Therefore, this study is designed to determine the prevalence of sacroiliitis and spondyloarthritis in patients diagnosed with sarcoidosis and to establish any possible correlation with the findings of the disease.

## 2. Materials and Methods

Forty-two consecutive sarcoidosis patients followed by our rheumatology outpatients clinic between December 2011 and June 2013 were enrolled in this study. The initial diagnosis of sarcoidosis had been made by the rheumatologists with reference to clinical signs and symptoms and histopathological confirmation of noncalcified granulomas in the biopsies of various organs and tissues. Conditions that might cause granulomatous disease (such as bacterial and fungal infections) had been ruled out. These patients had the diagnosis of sarcoidosis when enrolled in the study. After given consents were taken, laboratory tests were performed in all patients, including routine biochemistry, acute phase reactants (ESR, CRP), serum angiotensin converting enzyme (ACE), and calcium and hydroxyvitamin D3 levels. HLA-B27 typing was performed with the PCR method. Thoracic computerized tomography was performed for staging of sarcoidosis. Complete anamnesis was taken in detail, and systemic and rheumatologic examinations were performed. Detailed questioning in regard to spondyloarthritis (the existence of inflammatory back pain, gluteal area pain, uveitis, enthesitis, peripheral arthritis, dactylitis, psoriasis, inflammatory bowel disease, the presence of a preceding infection, and family history for spondyloarthritis) was performed. The presence of IBP was judged according to the Calin [11] criteria. The diagnoses of spondyloarthritis were made based on the European Spondyloarthritis Study Group (ESSG) [12] and the Assessment of Spondyloarthritis International Society (ASAS) classification criteria [13]. Lateral heel radiographs were performed to assess the enthesitis. Standard pelvic radiographs were obtained in all patients to assess the sacroiliac joints (SIJs). Each SIJ was scored on radiographs according to the mNY criteria [14] as follows: grade 0: normal; grade 1: suspicious; grade 2: minimal abnormality with small localized erosions, sclerosis without joint spondyloarthritis alteration; grade 3: definite abnormality with erosion, sclerosis, and joint spondyloarthritis widening or narrowing or partial ankylosis; grade 4: total ankylosis of joint. When/if sacroiliitis was determined in radiography, magnetic resonance imaging of SIJ was performed using the short time inversion recovery (STIR) method for the confirmation of the existence of active sacroiliitis. Radiological imaging was evaluated by a radiologist experienced in musculoskeletal system imaging.

### 2.1. Statistical Analysis

Crosstabs were produced to analyse the data, and chi-square analysis or Mann-Whitney* U* testing was performed as appropriate. 0.05 was taken as the threshold level of statistical significance.

## 3. Results

Forty-two sarcoidosis patients (ten males, thirty-two females) were included in the study. The mean age was 45.2 years (from 20 to 70 years), and mean duration of the illness was 3.5 years. When the manifestations of sarcoidosis were reviewed, it has been observed that twenty patients (47.6%) had erythema nodosum, three patients (7.1%) had acute, unilateral, anterior, and nongranulomatous uveitis, one patient (2.3%) had myositis, one patient (2.3%) had neurosarcoidosis, and thirty-two patients (76.2%) had arthritis. Twenty-eight of the patients with arthritis (87.5%) had ankle involvement, three patients (9.4%) had knee joint involvement, and one patient (3.1%) had wrist involvement. None of the patients had cardiac involvement. The thoracic computerized tomography scans revealed that twelve patients (28.5%) had stage 1 sarcoidosis, twenty-two patients (52.4%) had stage 2 sarcoidosis, four patients (9.5%) had stage 3 sarcoidosis, and four patients (9.5%) had stage 4 sarcoidosis. Laboratory assessments revealed that fifteen patients (35.7%) had elevated serum ACE, six patients (14.3%) had elevated serum calcium, two patients (4.7%) had elevated serum D3, twenty-nine patients (69%) had elevated ESR, and thirty patients (71.4%) had elevated CRP.

**Figure 1:**
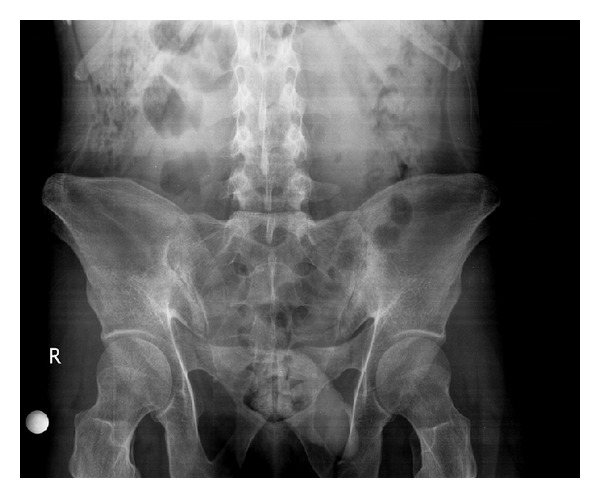
Direct radiography of the sacroiliac joints showing bilateral sacroiliitis.

Sacroiliitis was diagnosed in six of the forty-two (14.2%) sarcoidosis patients. All the patients with sacroiliitis were females. While the average age of the cases with sacroiliitis was 55 year, the average duration of disease was 17.8 months. When the first application symptoms of the patients with sacroiliitis were evaluated, it was observed that two patients applied with erythema nodosum, two patients applied with respiration symptoms, and two patients applied with locomotor system complaints. All the six patients complained of inflammatory low back pain. Different stages of sarcoidosis were diagnosed in each of the six patients: a stage 1 diagnosis was given in two patients, a stage 2 diagnosis in two patients, and a stage 4 diagnosis in two patients. In radiological staging of sacroiliitis, stage 2 sacroiliitis was found in 6 patients (Figure 1). When the patients were evaluated for HLA B-27, it was found negative in six patients. Comparison between patients with and without sacroiliitis revealed statistically significant differences in terms of some of the parameters (age, inflammatory back pain, enthesitis, and CRP levels) (Table 1). All the six patients with sacroiliitis were diagnosed with spondyloarthritis according to the criteria of ASAS (Assessment of Spondyloarthritis International Society) and of ESSG (European Spondyloarthropathy Study Group).

## 4. Discussion

Sarcoidosis may mimic a number of primary rheumatic diseases and/or coexist with them [15]. Involvement of the joints can be in 2 different ways; while acute, migratory, symmetric arthritis is more common, chronic recurrent erosive form can be especially rare [16]. Sacroiliitis is an important finding of the diseases of spondyloarthritis group, but many different causes (e.g., pyogenic, mycobacterial, and fungal infections and malign infiltrations) should also be eliminated. While the prevalence of spondyloarthritis is from 1% to 1.9% in the general population, Erb et al. reported prevalence of spondyloarthritis in patients with sarcoidosis as 6% [17]. In our study, the prevalence of sacroiliitis in cases diagnosed with sarcoidosis has been 14.2%. Comparisons revealed significant statistical differences in the patient group with sacroiliitis and the group without sacroiliitis, in terms of some of the parameters (age, inflammatory back pain, enthesitis, and CRP level). Furthermore, all the patients with sacroiliitis were also diagnosed with spondyloarthritis according to the ASAS and ESSG criteria. These findings suggest that both diseases may have a common etiopathogenesis and/or sacroiliitis may be an important clinical finding in patients with sarcoidosis. However, the etiopathogenesis of spondyloarthritis is not clear; it starts with an immunological reaction which develops against an undetermined bacterial antigen. Among the findings that support this theory are the DNA sequences of the many bacteria (i.e.,* Mycobacteria*,* Yersinia*, and* Salmonella*) found in patients with spondyloarthritis [18]. The etiology of sarcoidosis is unknown, but the possibility of chronic bacterial infection similar to spondyloarthritis is under investigation since the DNA of mycobacteria strains has been shown [19]. In the light of these discoveries, it is considered that infection-related reactive arthritis and sacroiliitis may develop in patients with sarcoidosis with immune susceptibility.

Different genetic factors may be involved in the development of sarcoidosis and spondyloarthritis. The role of the molecule HLA-B27 is obvious in the pathogenesis of spondyloarthritis and the elevated molecule HLA-DR5 has been found in patients with sarcoidosis [20]. Furthermore, it has been suggested that the HLA-B8 and HLA-DR3 molecules are related to acute sarcoid arthritis and spontaneous remission [21]. The fact that the two diseases develop on different genetic bases (MSC class 1 in spondyloarthritis and MHC class 2 in sarcoidosis) suggests a coincidence rather than a common etiopathogenesis. Elevated quantity of CD4+ T lymphocytes has been found in both diseases. Some agents (e.g., Propionibacterium acnes) have been detected in tissue samples in both diseases, but their role in pathogenesis has not yet been determined [22]. Another study has discovered that spondyloarthritis finding has been determined in 13.6% of HLA-B27-positive patients; the study has reported that there is elevated risk of spondyloarthritis with this allele [23]. Furthermore, while bilateral sacroiliitis is seen in HLA-B27-positive patients, unilateral sacroiliitis was found in HLA-B27-negative patients. A further study has recorded three HLA-B27-positive patients among fifteen cases determined to have sarcoidosis spondyloarthritis [24]. In our study, all the cases determined to have sacroiliitis were also HLA-B27-negative. This suggests that different mechanisms may be involved in the pathogenesis of sacroiliitis developing in patients with sarcoidosis.

This study has some limitations in the detection of sacroiliitis. Firstly, the diagnosis of sacroiliitis was performed using X-radiography in the first instance and further confirmed by MRI of SIJ. However, if MRI was performed in all patients, higher prevalence of sacroiliitis could have been detected at earlier stages of the disease. But we are conscious that the MRI method is too expensive, and the use of X-radiography for the small number of patients was considered cost-effective. Secondly, the number of patients was small in our study and it will be wrong to make a generalization.

In conclusion, we detected a higher prevalence of sacroiliitis in patients with sarcoidosis when compared to the general population. Patients with sarcoidosis should be questioned in terms of inflammatory back pain and should be assessed with SIJs imaging and/or MRI. Trials that involve large series of patients are needed for this.

## Figures and Tables

**Table 1:** Clinical, laboratory, and demographic characteristics of sarcoidosis patients with (*N* = 6) and without (*N* = 36) sacroiliitis.

Parameter	Patients with sacroiliitis (% of total)	Patients without sacroiliitis (% of total)	*P* value
Age, mean (year)	55	43.7	0.046
Gender (female)	6 (100%)	26 (72.2%)	0.308
Disease duration (month)	17.8	41.3	0.800
Inflammatory back pain	6 (100%)	8 (22.2%)	0.001
Enthesitis	6 (100%)	6 (16.7%)	0.000
Erythema nodosum	5 (83.3%)	15 (41.7%)	0.087
Uveitis	0 (0%)	3 (8.3%)	1.000
Ankle arthritis	6 (66.7%)	22 (61.1%)	1.000
Elevated serum ACE level	2 (33.3%)	13 (38.9%)	1.000
Elevated serum calcium level	2 (33.3%)	4 (11.1%)	0.197
Elevated serum D3 level	0 (0%)	2 (5.6%)	1.000
HLAB27 positive	0 (0%)	2 (5.6%)	1.000
Elevated ESR	3 (50%)	26 (72.2%)	0.353
Elevated CRP	2 (33.3%)	28 (77.8%)	0.046

ACE: angiotensin converting enzyme; ESR: erythrocyte sedimentation rate; CRP: C reactive protein.
